# The Malingering Problem

**Published:** 1913-07-05

**Authors:** 


### THE MALINGERING PROBLEM.

One of the most serious difficulties which threaten the finance of the Insurance Act is the detection and prevention of malingering. This question was one of those which were never seriously discussed when the Act was a Bill before Parliament, owing to the fatal haste with which all the proceedings had to be rushed from motives of party and political expediency. Now, however, there is no avoiding it, for facts are stubborn things, and successful malingering fairly spells ruin to some of the approved societies. First, there seems no doubt that malingering is vastly more prevalent among women than men. Hence societies composed wholly or mainly of women are feeling the pinch much more than those consisting wholly or mainly of male members. Exactly why this should be can be guessed at rather than proved; probably several reasons combine to favour it. But the evil of malingering is a desperately serious problem not only in approved societies composed of women but throughout the operations of the Act, and for that matter throughout the country. Observers well qualified to judge believe that the malingering problem was first created by the Workmen's Compensation legislation of one and two decades ago; and the Insurance Act has merely stimulated and fertilised this growth.

Social reformers—and every decent citizen realises that mid-Victorian conditions of life for the labouring classes were in most ways very unsatisfactory—are faced with a dilemma which impales them whenever they try to deal with the health of the proletariat. Either they must allow a percentage of the population to suffer real hardship owing to the hazard of sickness and accident, or else, when they seek to minimise this evil by Factory Acts, Compensation Acts, Compulsory Insurance, and the like, their action will result in rendering large sections of the population permanently work-shy. It is no use blinking this difficulty, as some of those who have discussed it are doing when they seek to lay the blame on the doctors. Doctors are human beings; they are working an Act which most of them do not like on terms which they do not think adequate. Some of the good men are on the panels; all the wasters are. Most of them are overworked. Is it likely that such a medical service can be trusted to check malingering, a feat which requires much time, skill, and patience? The most practical proposal hitherto suggested is for the appointment of medical referees, who shall themselves have nothing to do with the treatment of the insured. But this involves very considerable expense, which the Committees can ill afford. Still, it seems to be the only way out of a difficulty which ought to have been foreseen by Parliament, just as it was foreseen by medical men and friendly society officials. However great the temptation to malinger offered by the Act may be, we cannot condone the moral offence of those who succumb to it; and to assert, as one speaker at a recent conference did, that the stigma is greater on the doctor than on the malingerer seems to us simply disgraceful. Verily, the chickens of stifled discussion are coming home to roost!

